# Modelling the impact of adopting new-generation insecticide-treated nets on malaria transmission and insecticide resistance

**DOI:** 10.1186/s13071-026-07611-x

**Published:** 2026-08-14

**Authors:** Hamenyimana E. Gervas, Maranya M. Mayengo, Frank Chacky, Yeromin P. Mlacha, Halfan S. Ngowo, Fredros O. Okumu, Prashanth Selvaraj

**Affiliations:** 1 Nelson Mandela African Institution of Science and Technology, P.O. Box 447, Arusha, Tanzania; 2 Environmental Health and Ecological Science Department, Ifakara Health Institute, P.O. Box 53, Ifakara, Tanzania; 3 Department of Mathematics and Statistics, University of Dodoma, P.O. Box 338, Dodoma, Tanzania; 4 National Malaria Control Programme, P.O. Box 743, Dodoma, Tanzania; 5 School of Biodiversity, One Health and Veterinary Medicine, University of Glasgow, Glasgow, G12 8QQ UK; 6 Institute for Disease Modeling, Global Health Division, Gates Foundation, Seattle, WA USA

## Abstract

**Background:**

Widespread insecticide resistance increasingly threatens malaria elimination, prompting a reassessment of vector control strategies. As Tanzania transitions from standard pyrethroid-only insecticide-treated nets (ITNs) to new-generation nets, evaluating the impact of this shift on malaria transmission and resistance is critical.

**Methods:**

Using the agent-based malaria model, EMOD, we assessed the impact of three ITN types, standard pyrethroid-only nets, pyrethroid-PBO nets (Olyset® Plus/PermaNet® 3.0), and the dual-active Interceptor® G2 nets (IG2) on malaria transmission and on modelled changes in resistance genotype frequencies under a simplified multi-locus representation of insecticide resistance. We also evaluated different sequences for introducing the new-generation nets, and the impact of combining ITNs with indoor residual spraying (IRS). The model was calibrated using incidence and prevalence data from two regions in northwestern Tanzania, incorporating transmission seasonality and heterogeneity, insecticide resistance, and behaviours of dominant vectors *Anopheles funestus* (highly anthropophilic, endophilic) and *Anopheles arabiensis* (more opportunistic readily biting non-human hosts outdoors).

**Results:**

Changing from standard pyrethroid-only ITNs to pyrethroid-PBO and thereafter to IG2 ITNs reduced homozygous-resistant *An. funestus* and *An. arabiensis* by 71.9% (95% CI 70.8–72.9%) and 81.4% (95% CI 78.7–84.1%), respectively within the vector population, and reduced malaria incidence and prevalence by 63% (95% CI 51.0–74.5%) and 76% (95% CI 66.5–89.2%), respectively, by year nine. Deploying IRS before the peak malaria transmission season in mid-May, in the second year following pyrethroid-PBO ITNs distribution, and repeating this every three years, reduced malaria incidence and prevalence by 68.4% (95% CI 24.4–112.5%) and 73.9% (95% CI 35.4–112.4%), respectively, compared with pyrethroid-PBO ITNs alone.

**Conclusions:**

In contrast to continuous use of standard pyrethroid-only ITNs, which sustains resistance selection, transitioning to new-generation ITNs, with or without periodic IRS, may disrupt the evolutionary trajectory of pyrethroid resistance, reduce malaria burden, and strengthen progress towards elimination.

**Graphical Abstract:**

**Figure:**
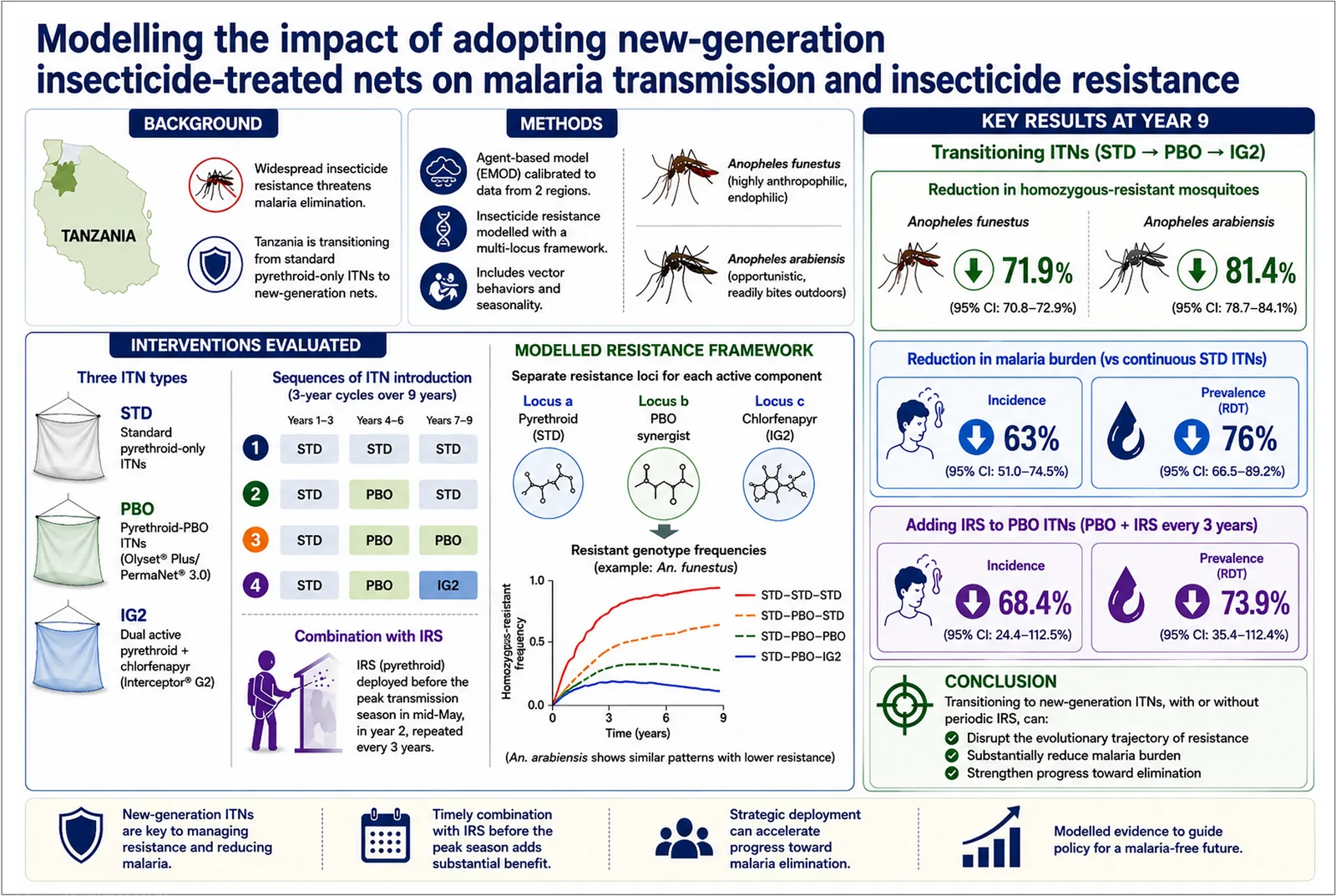

**Supplementary Information:**

The online version contains supplementary material available at https://doi.org/10.1186/s13071-026-07611-x.

## Background

Malaria vector control has expanded significantly since 2000, primarily through insecticide-treated nets (ITNs) and indoor residual spraying (IRS) as core insecticide-based interventions [1]. Since their inception, these interventions, together with improved case management, have prevented an estimated 2.2 billion malaria cases and 12.7 million deaths [2, 3]. Despite being preventable and treatable, malaria remains a major global health challenge, with over 90% of the burden reported in sub-Saharan Africa [4, 5], where the recent increase in malaria cases is driven in part by rising resistance to antimalarial drugs [6, 7] and insecticides [8].

In particular, widespread insecticide resistance, reported in over 87% of malaria-endemic countries, poses a major obstacle to malaria elimination [4, 9]. The dual use of pyrethroids in agriculture and public health has been a major driver of insecticide resistance [10–13]. In response, the World Health Organization (WHO) has endorsed next-generation insecticide-treated nets that combine pyrethroids with either an enzyme synergist or a second insecticide with an alternative mode of action [14]. Examples of these new-generation ITNs include pyrethroid-piperonyl butoxide (PBO) nets (e.g. Olyset® Plus, containing permethrin and PBO, and PermaNet® 3.0, containing deltamethrin and PBO) as well as dual-active ingredient nets such as Interceptor® G2 (IG2 ITNs), which combines the pyrethroid alpha-cypermethrin with the pyrrole chlorfenapyr [9, 14].

In Tanzania, insecticide resistance especially to pyrethroids has been detected in 22 of the 62 vector surveillance sentinel sites and confirmed in all major malaria vectors (*Anopheles gambiae *sensu stricto (s.s.), *Anopheles arabiensis*, and *Anopheles funestus*), with resistance in these species primarily mediated not only by metabolic detoxification enzymes but also by multiple target-site mutations [15–18]. Persistent resistance remains one of the major barriers to achieving the national target of reducing malaria incidence and mortality by at least 90% by 2030 [19]. In response to this challenge, the National Malaria Control Programme (NMCP) has previously implemented non-pyrethroid IRS using organophosphate or neonicotinoid insecticides in few councils in the lake zone, replacing the previously used pyrethroid IRS [19–21]. However, following the recent discontinuation of IRS [22, 23], the NMCP, in collaboration with the US President Malaria Initiative (PMI) and Global Fund (GF), has been distributing pyrethroid-PBO ITNs (Olyset® Plus and PermaNet® 3.0) in areas previously reliant on IRS and standard pyrethroid-only ITNs mainly through a targeted mass campaigns every three years, while also deploying IG2 ITNs in few districts with the highest levels of resistance [22, 23].

Despite ongoing initiatives, the impact of transitioning from standard pyrethroid-only ITNs to pyrethroid-PBO and IG2 ITNs on malaria transmission and insecticide resistance dynamics remains insufficiently understood, particularly in areas previously protected by standard ITNs and IRS. Although previous modelling studies have substantially improved understanding of insecticide resistance management and the impact of standard and next-generation ITNs [24–28], many simplify resistance as a single-gene process or do not explicitly represent how different insecticides impose selection over time. Growing evidence indicates that insecticide resistance is often a complex polygenic trait involving multiple interacting genetic mechanisms [27, 29–33], with resistance evolution influenced by changing selection pressure, insecticide decay, and cross-resistance. Consequently, there remains a need for modelling frameworks that capture how transitions between insecticides with different modes of action redistribute selection across multiple resistance loci while simultaneously evaluating their effects on malaria transmission. Therefore, this study used the agent-based malaria model, EMOD [24, 25] to simulate the transition from standard pyrethroid-only ITNs to pyrethroid-PBO and IG2 ITNs in Tanzania. The model incorporates species-specific transmission dynamics, seasonal malaria transmission, and insecticide-specific selection acting on multiple resistance loci representing resistance to pyrethroids, PBO, and chlorfenapyr. Rather than attempting to capture the full quantitative-genetic architecture of insecticide resistance, we used EMOD’s vector genetics framework to represent resistance as a small number of chemistry-specific phenotypes. This approach enables evaluation of how changes in ITN active ingredients alter selection pressure on resistance and, consequently, malaria transmission outcomes.

## Methods

### EMOD malaria transmission model

In this study, all simulations were performed using the agent-based mechanistic model of malaria transmission (EMOD v2.20) [24, 25], which represents individual humans and mosquitoes and their interactions. The model integrates all four developmental stages of mosquito species: eggs, larvae, pupae, and adult mosquitoes, as local mosquito population dynamics are primarily driven by the availability of larval habitats [34]. The availability of larval habitats at any given time determines the number of larvae each habitat type can support, which consequently influences the number of adult vectors that emerge at the end of the life cycle [35, 36].

### Insecticide resistance

In EMOD, mosquito genetics are represented through inherited alleles, and insecticide resistance is implemented by mapping genotype classes to phenotypic modifiers of intervention effects [24, 25]. Thus, resistance evolution in the model occurs through differential survival: mosquitoes whose genotypes confer lower susceptibility to an insecticide are more likely to survive exposure and contribute to subsequent generations.

In this study, we used a simplified multi-locus representation of insecticide resistance. Resistance to each active chemistry was represented by an independent locus: locus *a* for pyrethroid resistance, locus *b* for reduced response to PBO-mediated restoration of pyrethroid susceptibility, and locus *c* for chlorfenapyr resistance. Pyrethroid-PBO ITNs therefore acted through loci *a* and *b*, whereas Interceptor® G2 ITNs acted through loci *a* and *c.* Each locus comprised three genotype classes: homozygous susceptible, heterozygous, and homozygous resistant. Heterozygotes were assumed to exhibit partial resistance, while the two homozygotes represented fully susceptible and fully resistant phenotypes, respectively [25, 37–39].

These loci should be interpreted as aggregate resistance phenotypes associated with each chemistry, not as specific molecular loci or single target-site mutations. The model therefore captures chemistry-specific selection and propagation of resistance phenotypes during transitions between ITNs with different modes of action, but it does not explicitly represent the full polygenic architecture of insecticide resistance, including multiple target-site mutations, metabolic detoxification pathways, cuticular resistance, epistasis, compensatory mutations, or the emergence of new resistance mechanisms.

A more explicitly polygenic representation could in principle be configured in EMOD [24] by defining multiple loci for each insecticide chemistry and mapping multi-locus genotypes, or allele-count score classes, to graded killing modifiers. Such an approach would approximate a discrete Polygenic Resistance Score [27]. However, it would require additional data on initial allele frequencies across loci, dominance and epistatic effects, genotype-to-phenotype survival relationships, fitness costs, cross-resistance among chemistries, and longitudinal changes in resistance phenotypes or genotypes. These data were not available for the study settings. We therefore used a parsimonious multi-locus representation designed to compare relative selection pressures under alternative ITN deployment strategies.

### Characterization of mosquito species

In Tanzania, *Anopheles gambiae* s.s., *An. arabiensis*, and *An. funestus* are primary malaria vectors. Although *An. gambiae* s.s. contributes to malaria transmission in some settings [40], widespread insecticide use, particularly through ITNs, has shifted vector composition, with *An. arabiensis* and *An. funestus* now dominating in many regions [40–44]. Given their current epidemiological significance and resistance profiles [19, 42, 45], this study modelled vector populations using *An. arabiensis* and *An. funestus*, assigning indoor feeding/resting fractions of 63.1% and 78.2% [46], respectively, with indoor feeding and resting assumed equal. These species were further assigned anthropophilic indices of 44% [47] and 64% [48], respectively. Seasonality was modelled based on observed monthly variations in mosquito abundance from lake zone in Tanzania [43]. The adult life expectancy values represent EMOD baseline adult survival parameters rather than direct estimates of realized field lifespan. With vector ageing enabled, EMOD applies age-dependent senescence, resulting in a shorter realized adult lifespan than the nominal parameter value [49]. Baseline mosquito ecological parameters, including life expectancy, anthropophily, and indoor feeding behaviour, were assumed to remain constant throughout the simulations, whereas the effects of ITNs and IRS on mosquito mortality, host-seeking success, and blood-feeding were modelled dynamically through the intervention modules.

### Model calibration

**Fig. 1:**
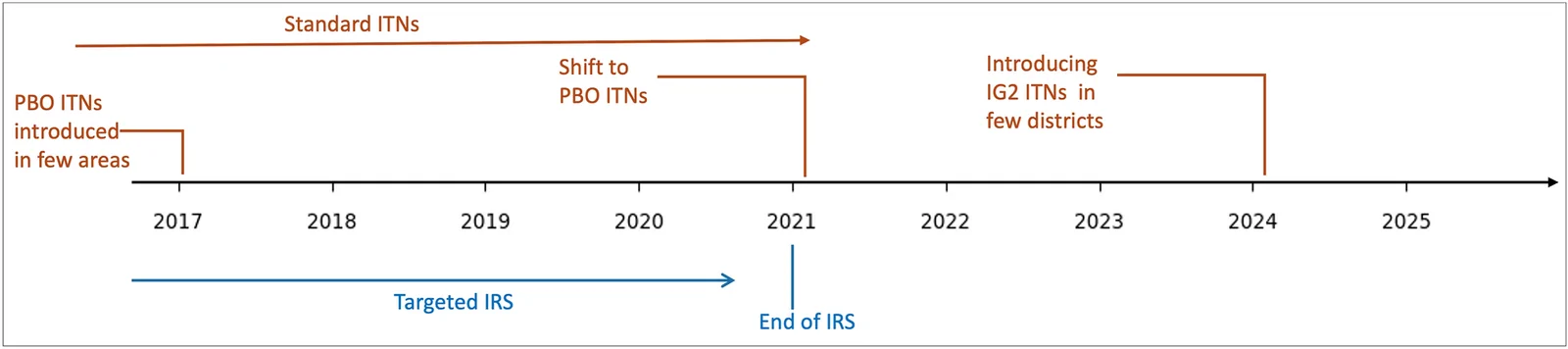
Timeline of ITN and IRS interventions in Tanzania (2017–2025) illustrating the transition from standard pyrethroid-only ITNs (standard ITNs) to new-generation ITNs [19, 51]

In Tanzania, ITNs serve as the primary malaria intervention and have been widely distributed nationwide since 2007 [50]. Since then, significant efforts have been made to enhance ITN coverage through various distribution channels, including mass campaigns, school net programmes, antenatal care, and targeted replacement campaigns [50]. Following the cessation of IRS activities in 2021, the country has been undergoing a phased transition, beginning with the replacement of standard pyrethroid-only ITNs with pyrethroid-PBO ITNs, followed by the deployment of IG2 ITNs in selected districts [23] (refer to Fig. 1 for the timeline of ITNs and IRS).

**Fig. 2:**
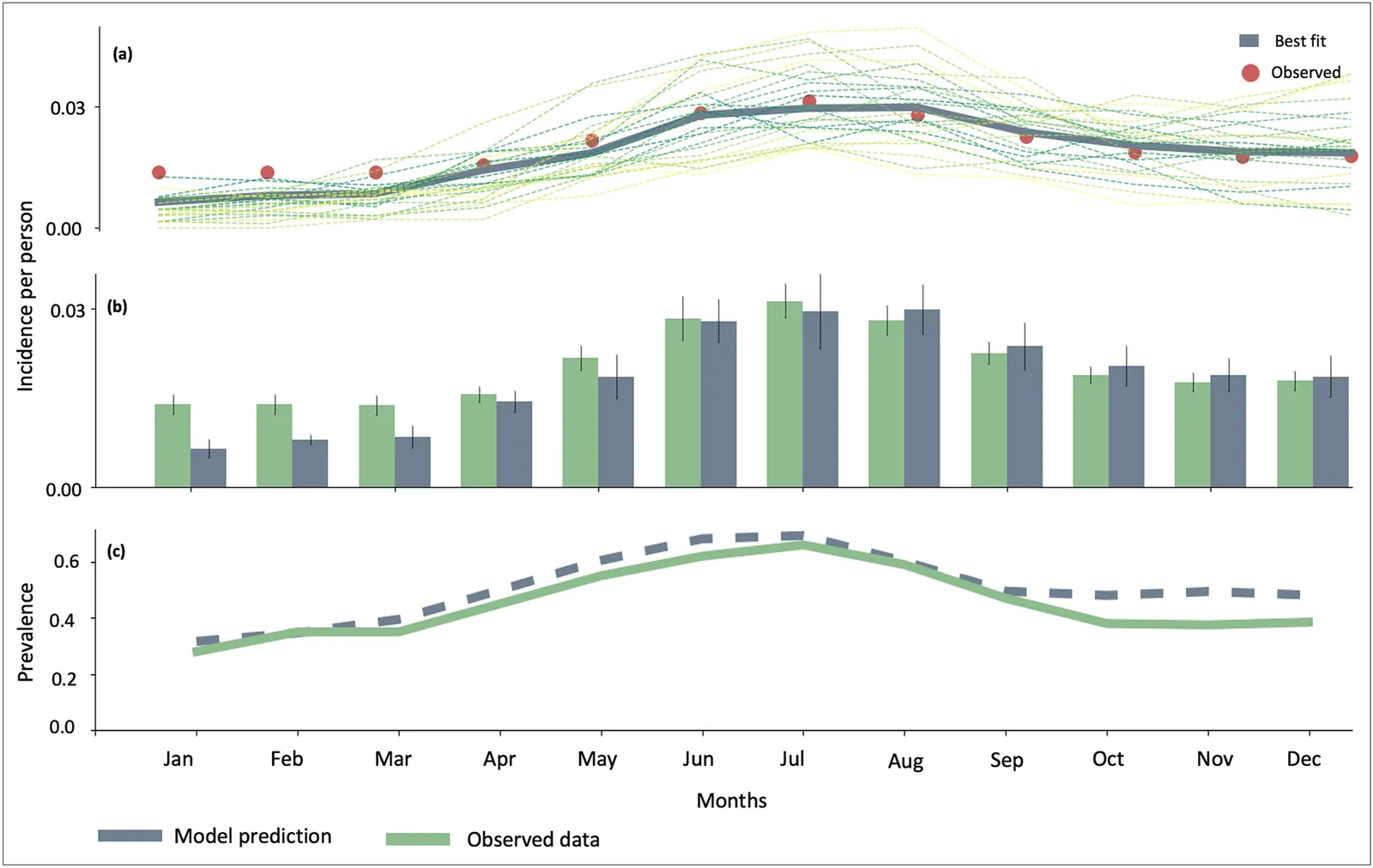
Model calibration: **a** the dashed lines represent model realizations with relatively low mean squared errors; the grey curve indicates the best fit; **b** best-fit versus observed incidence cases; **c** model prediction versus observed prevalence. The incidence data were calibrated using 2019 data from the four districts of Mwanza region [53], while prevalence was calibrated using 2020 data from five districts of Geita region [52]

The model was calibrated using the 2020 monthly prevalence data from five councils in Geita Region, Tanzania (Bukombe, Chato, Mbogwe, Nyang’hwale, and Geita District Councils) [52], and the 2019 monthly incidence data from four councils of Mwanza region (Misungwi, Ukerewe, Magu, and Kwimba) [53]. These councils represent the lake zone of Tanzania, characterized by widespread insecticide resistance, prior IRS implementation, and the ongoing transition to new-generation nets. The model was calibrated by adjusting the monthly larval habitat parameters for *An. funestus* and *An. arabiensis* to reproduce observed transmission patterns. For each larval habitat parameter set, simulation outputs were compared with field data using mean squared error (MSE). The best-fit curve was obtained by taking the unweighted average of the outputs from the 10 simulations with the lowest MSE values among all calibration runs, resulting in a final MSE of 0.0008 (Fig. 2). During calibration, a cohort of 1000 individuals was simulated over 40 years to establish age-specific immunity. The incidence dataset was not stratified by age, whereas the prevalence data specifically represented children aged 0–5 years. The model reproduces the observed seasonal dynamics, capturing the peak malaria transmission (Fig. 2).

### Modelled interventions

This study evaluated the impact of standard pyrethroid-only, pyrethroid-PBO, and IG2 ITNs on malaria transmission and insecticide resistance dynamics. It further assessed the strategic deployment of organophosphate-based IRS in combination with pyrethroid-PBO ITNs. No additional interventions were considered. All interventions target adult female mosquitoes during host seeking and resting, with a uniform population coverage of 80%. ITNs were distributed every three years, typically before the onset of the peak malaria transmission season in March (Fig. 2). This timing was chosen to represent a typical deployment schedule for simulation purposes and may not necessarily reflect current ITN distribution practices in Tanzania.

### Sensitivity analysis

To assess the robustness of the model predictions, we performed one-at-a-time sensitivity analyses by varying each parameter in Table 1 individually from 0.6 to 1.4 times its nominal value (± 20% and ± 40%), while holding all other parameters constant. Probability and fractional parameters were capped at 1.0 where applicable. The analyses considered repeated deployment of standard pyrethroid-only ITNs, sequential transition from standard pyrethroid-only ITNs to pyrethroid-PBO and IG2 ITNs, and a targeted analysis of the IRS-specific parameters for a pyrethroid-PBO ITN programme combined with IRS in year 2.

Detailed descriptions and sources of all model parameters are provided in Appendix, Table 1. Additional details on parameter derivation, together with the sensitivity analysis methods and results, are provided in the Supplementary Material (Figures SA1 and SA2; Tables SA1, SA2, SA3, and SA4).

## Results

We conducted a two-stage scenario analysis. First, we compared standard pyrethroid-only ITNs with new-generation ITNs (pyrethroid-PBO and IG2). Second, the impacts of ITN transition (sequential deployment strategies) on malaria transmission and the evolution of insecticide resistance were evaluated. Each scenario was replicated across 30 independent stochastic realizations to account for random variability and generate robust estimates of intervention impacts. Numerical results are presented as the mean across 30 stochastic realizations, with uncertainty quantified using 95% confidence intervals (mean ± 1.96 × SD/√30).

### Impact of pyrethroid-PBO and IG2 ITNs on malaria transmission compared to standard pyrethroid-only ITNs

The simulation was conducted over a 3-year period, with ITN deployment assumed to occur at the start of the peak malaria season in March (Fig. 2).

**Fig. 3:**
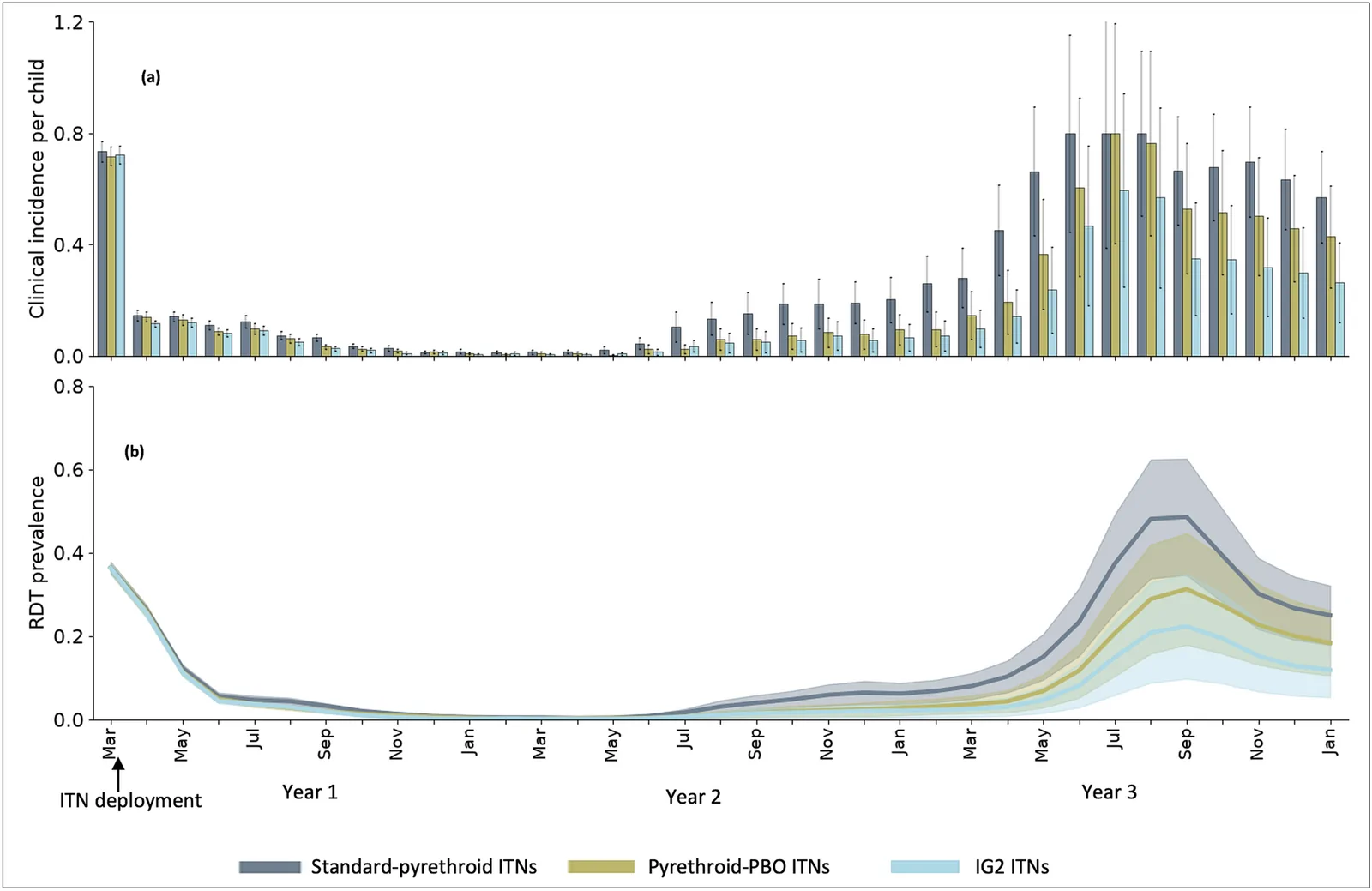
The impact of pyrethroid-PBO ITNs and IG2 ITNs on **a** malaria incidence and **b** prevalence in comparison with standard pyrethroid-only ITNs for children less than 5 years. Each type of ITN was assumed to be deployed at start of the peak malaria season in March. Error bars and shaded regions indicate 95% confidence intervals

Figure 3 shows the comparison of the impact of standard pyrethroid-only, pyrethroid-PBO, and IG2 ITNs on malaria clinical incidence and RDT prevalence in children under 5 years of age. Throughout the three-year period, IG2 ITNs achieved the greatest reduction in malaria burden, followed by pyrethroid-PBO ITNs. During the third year, IG2 ITNs reduced clinical incidence by 54.8% (95% CI 22.4–87.2%) relative to standard pyrethroid-only ITNs. Pyrethroid-PBO ITNs were associated with an estimated reduction of 33.7% (95% CI − 5.7 to 73.0%), although the confidence interval included zero, indicating considerable uncertainty. Similarly, IG2 ITNs reduced RDT prevalence by 56.4% (95% CI 24.6–88.3%), whereas pyrethroid-PBO ITNs were associated with an estimated 37.2% reduction (95% CI − 0.6 to 75.0%), with the confidence interval likewise including zero. Despite these third-year reductions, clinical incidence and RDT prevalence remained substantial, suggesting that ITNs alone are unlikely to sustain malaria control and highlighting the need for complementary interventions.

**Fig. 4:**
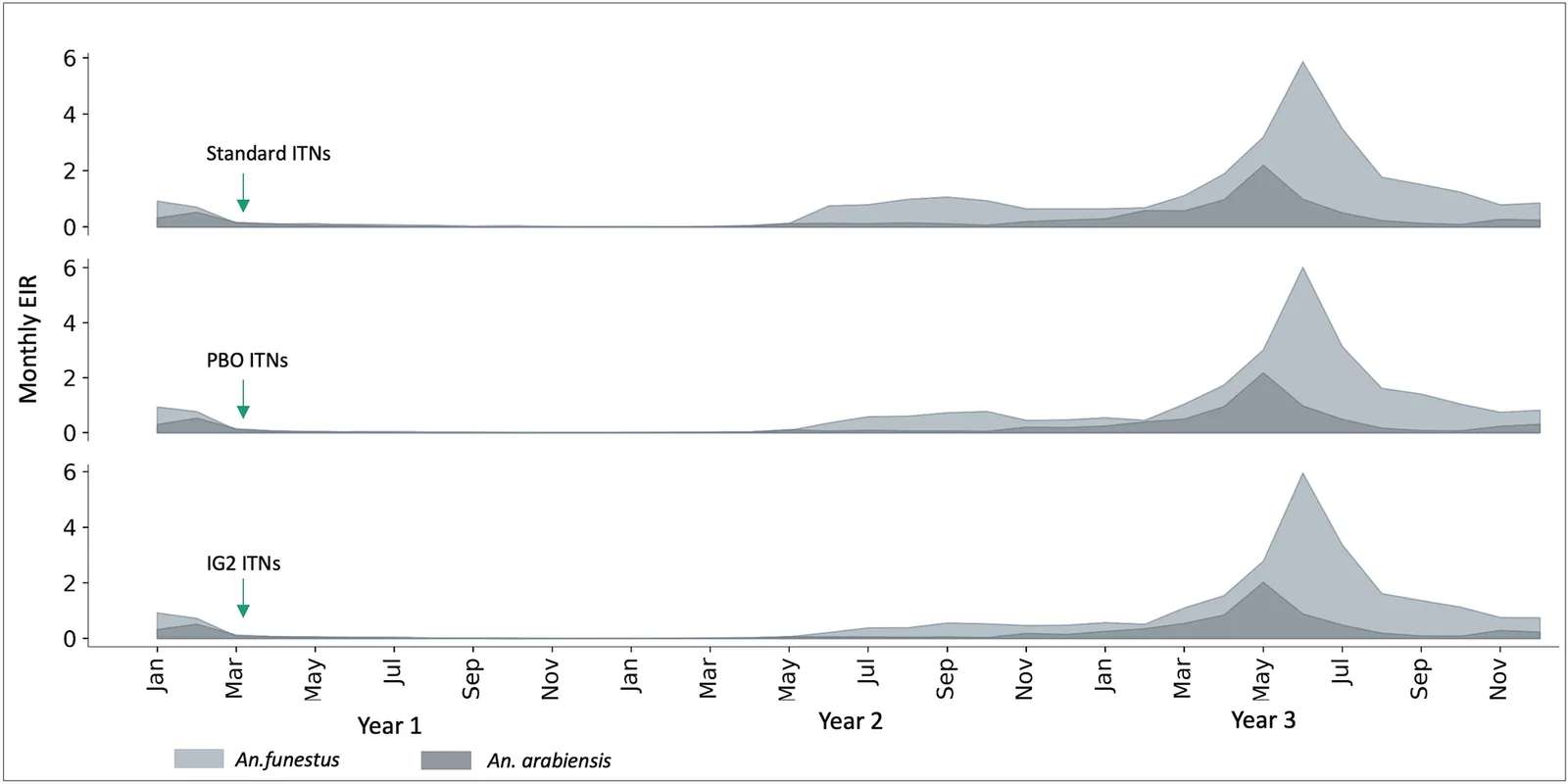
The impact of ITNs on monthly EIR. *An. arabiensis* and *An. funestus* were assigned indoor feeding/resting fractions of 63.1% and 78.2% [46], and anthropophilic indices of 44% [47] and 64% [48], respectively. Standard ITNs refer to pyrethroid-only ITNs, PBO ITNs to pyrethroid-PBO ITNs, and IG2 to Interceptor® G2

**Fig. 5:**
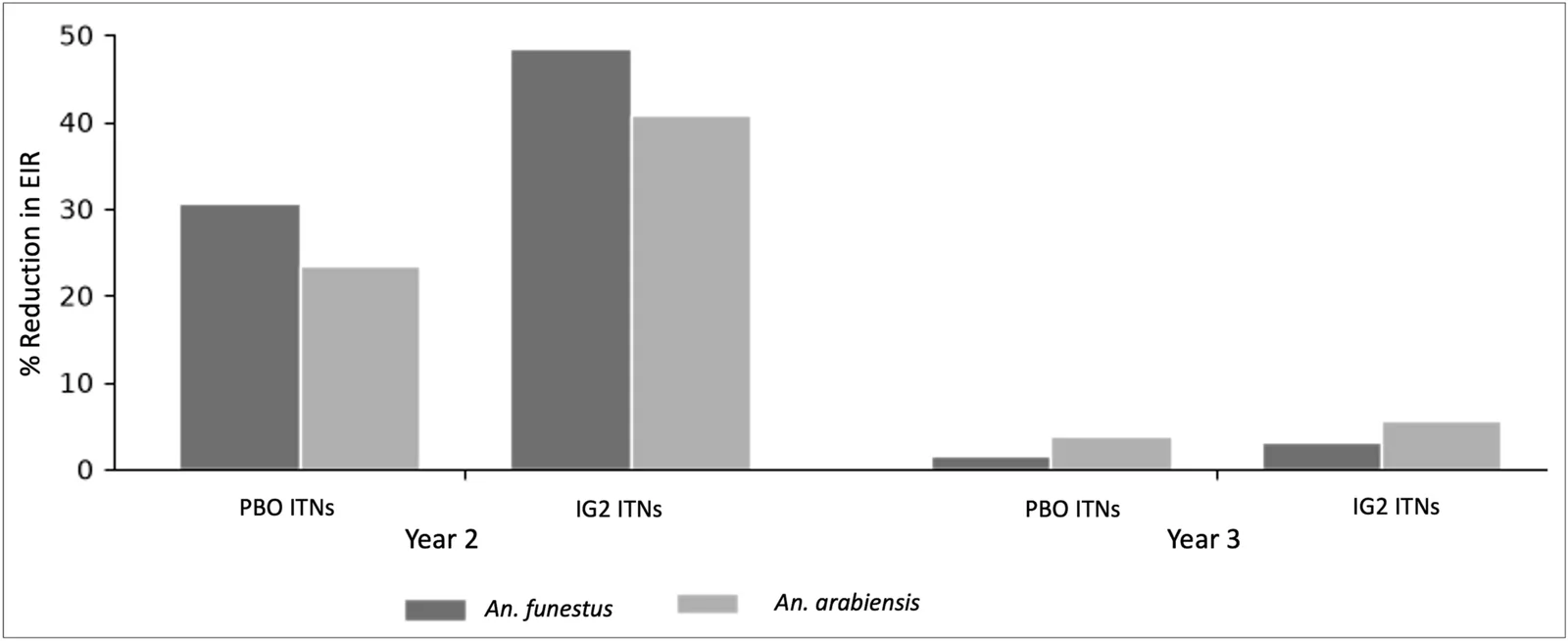
The percentage reduction in EIR relative to standard pyrethroid-only ITNs during year 2 and 3. PBO ITNs refer to pyrethroid-PBO ITNs, and IG2 to Interceptor® G2 ITNs. Percentage reductions are with respect to yearly average EIR values

Figures 4 and 5 demonstrate the impact of ITNs on monthly entomological inoculation rate (EIR), a measure of malaria transmission intensity. Compared with standard pyrethroid-only ITNs, IG2 ITNs produced the greatest reduction in EIR, decreasing monthly EIR by approximately 48% in *An. funestus* and 40% in *An. arabiensis* during the second year, compared with reductions of about 30% and 23%, respectively, achieved by pyrethroid-PBO ITNs. However, the reductions in EIR achieved by both ITN types declined by the third year, with slightly smaller reductions observed for *An. funestus* than for *An. arabiensis*. These findings highlight the need for complementary malaria control interventions beyond ITNs alone.

### Impact of integrating IRS with pyrethroid-PBO ITNs

The impact of a single IRS implementation over a three-year period, implemented before the peak malaria transmission season in mid-May (at the end of rain season in the lake zone [54]), was simulated during the second or third year following the deployment of pyrethroid-PBO ITNs.

**Fig. 6:**
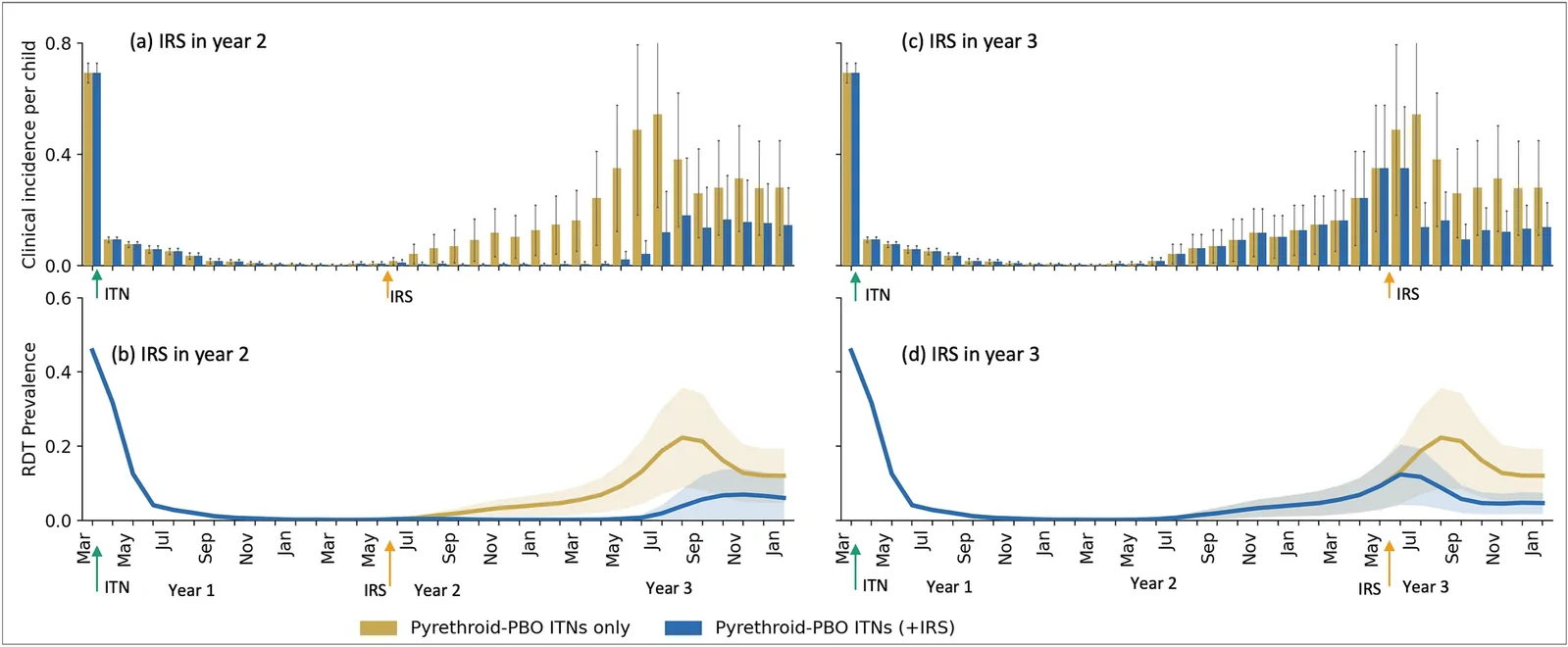
Impact of adding a single round of IRS every three years to pyrethroid-PBO ITN campaigns on malaria incidence and prevalence among children under five years of age. **a** and **b** IRS deployed before the peak transmission season in mid-May of the second year; **c** and **d** deployment in the third year. ITNs were distributed in March at the onset of the peak transmission season, with IRS applied in mid-May following the rainy season [54]. Error bars and shaded regions indicate 95% confidence intervals

**Fig. 7:**
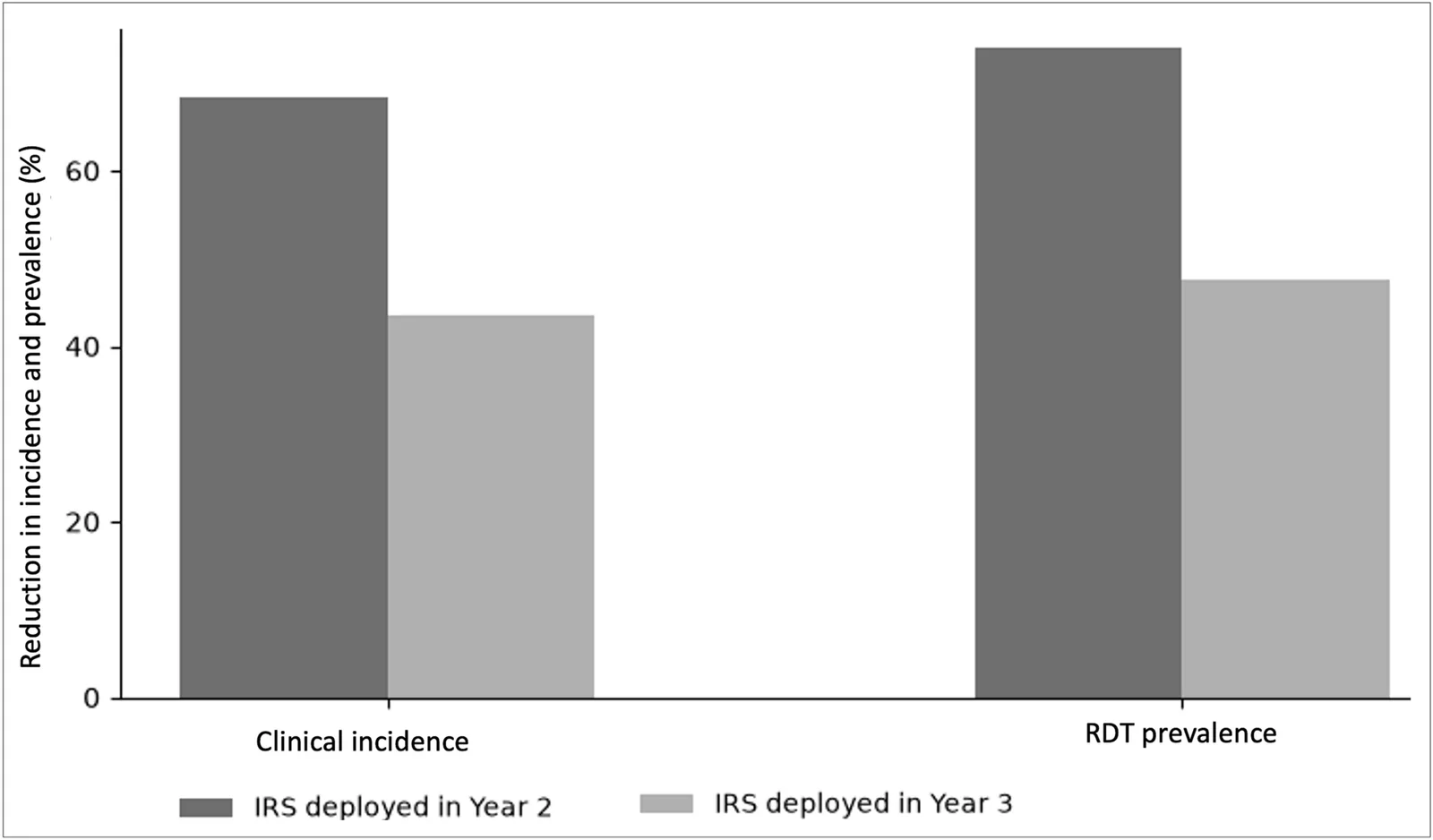
Percentage reduction in clinical incidence and RDT prevalence following the addition of a single round of IRS every three years to pyrethroid-PBO ITNs, relative to pyrethroid-PBO ITNs alone. Reductions were calculated from the cumulative clinical incidence and RDT prevalence over the entire third year following ITN deployment

Figures 6 and 7 illustrate the impact of supplementing pyrethroid-PBO ITNs with a single round of IRS every three years to counteract the decline in ITN effectiveness. When IRS is deployed before the peak malaria transmission season in the second year, the combined intervention reduces clinical incidence and RDT prevalence in the third year by 68.4% (95% CI 24.4–112.5%) and 73.9% (95% CI 35.4–112.4%), respectively, compared with pyrethroid-PBO ITNs alone. In contrast, deploying IRS before the peak transmission season in the third year results in smaller reductions of 43.6% (95% CI 11.1–98.3%) in clinical incidence and 47.5% (95% CI − 3.3–98.2%) in RDT prevalence (Fig. 7). Although malaria resurges during the third year under both strategies, IRS deployment in the second year outperforms deployment in the third year by providing more sustained reductions in malaria burden.

### Impact of changing from standard pyrethroid-only nets to new-generation ITNs

**Fig. 8:**
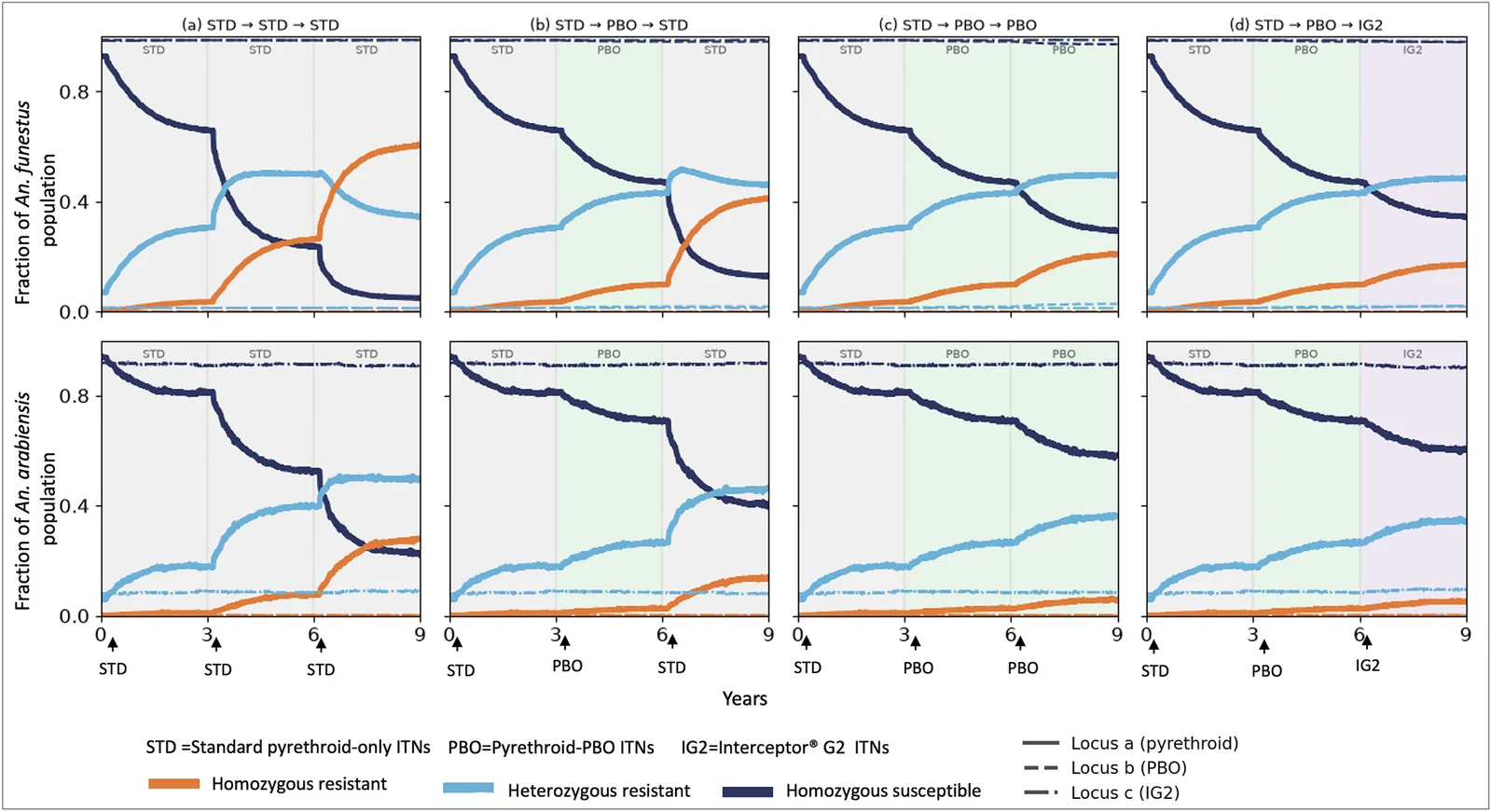
Impact of ITN transition on vector resistance genotype dynamics compared with the repeated use of standard pyrethroid-only ITNs. Shaded backgrounds indicate periods during which ITNs were active. The trajectories shown correspond to resistance loci (a, b, and c) associated with standard pyrethroid-only ITNs, pyrethroid-PBO ITNs, and Interceptor® G2 ITNs

To investigate how ITN transitions influence insecticide resistance evolution, four ITN deployment strategies were simulated over a 9-year period, with ITNs replaced every three years (Fig. 8). For pyrethroid-PBO and IG2 ITNs, mosquito mortality is assumed to result from the combined effects of their two active ingredients: pyrethroid and PBO for pyrethroid-PBO ITNs, and pyrethroid and chlorfenapyr for IG2 ITNs. Within the model [24, 25], changes in resistance genotype frequencies arise from differential survival following insecticide exposure. Mosquitoes carrying genotypes associated with reduced susceptibility to the active ingredient(s) are more likely to survive contact with ITNs and contribute to subsequent generations. Because pyrethroid, PBO-mediated synergistic activity, and chlorfenapyr decay at different rates, their relative contributions to mosquito mortality change over time, producing temporal shifts in modelled selection pressure.

#### Repeated deployments of standard pyrethroid-only ITNs

**Fig. 9:**
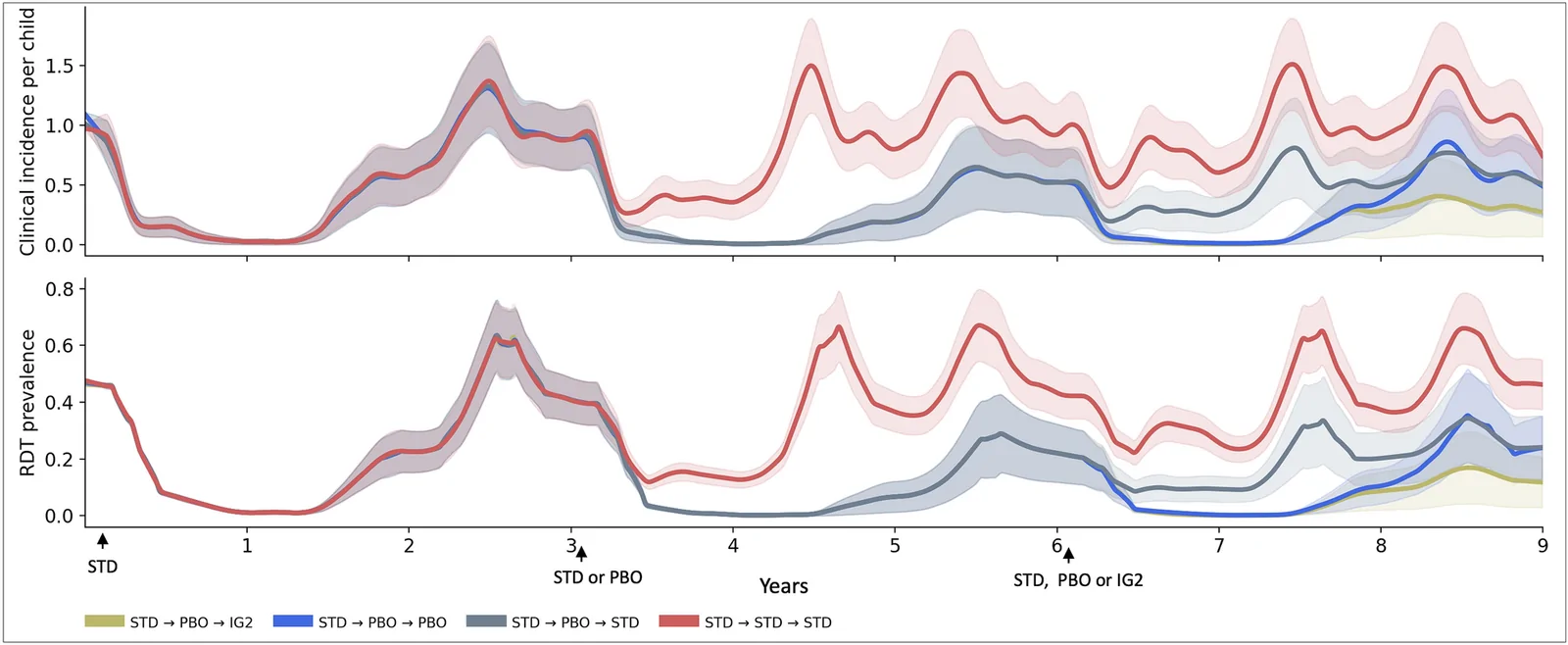
The impact of ITN transition: **a** incidence, **b** prevalence for children under five, compared to repeated deployments of standard pyrethroid-only ITNs. ITNs are distributed triennially, commencing at the at start of the peak malaria season on March, each at 80% coverage, shaded regions indicate 95% confidence intervals

Continuous deployment of standard pyrethroid-only ITNs (Fig. 8a) imposed sustained selection on the pyrethroid resistance locus (*a*). Pyrethroid-susceptible mosquitoes experienced high mortality, resulting in a rapid decline in homozygous susceptible genotypes and a progressive increase in heterozygous and homozygous-resistant mosquitoes, particularly in *An. funestus*. Repeated exposure to the same insecticide continuously favoured mosquitoes carrying the resistant allele, leading to homozygous-resistant frequencies of 0.6 (95% CI 0.598–0.61) in *An. funestus* and 0.28 (95% CI 0.26–0.29) in *An. arabiensis* by year nine. Consequently, continued use of the same ITNs also sustained a high malaria burden (Fig. 9, red curve).

#### Rotational deployments of standard pyrethroid-only ITNs and pyrethroid-PBO ITNs

Replacing standard pyrethroid-only ITNs with pyrethroid-PBO ITNs (Fig. 8b) reduced the rate of pyrethroid resistance accumulation. During the PBO deployment phase, mosquito mortality resulted from both pyrethroid and the PBO-mediated restoration of pyrethroid susceptibility, reducing the survival advantage of pyrethroid-resistant mosquitoes. Consequently, selection shifted partially from the pyrethroid resistance locus (*a*) towards the PBO-associated locus (*b*). As PBO efficacy declined more rapidly than pyrethroid efficacy, selection gradually reverted towards pyrethroid resistance before the subsequent reintroduction of standard pyrethroid-only ITNs intensified selection at locus *a*. Compared with continuous standard pyrethroid-only ITNs deployment, this strategy reduced homozygous-resistant frequencies by 32.0% (95% CI 30.4–33.5%) in *An. funestus* and 51.3% (95% CI 45.6–56.9%) in *An. arabiensis* by year 9. It also reduced clinical malaria incidence by 39% (95% CI 26.2–49.1%) and prevalence by 43.5% (95% CI 33.0–51.2%) (Fig. 9, grey graph).

#### Transitioning from standard pyrethroid-only ITNs to pyrethroid-PBO ITNs

Extending pyrethroid-PBO deployment for 6 years (Fig. 8c) further reduced continuous selection on the pyrethroid resistance locus. The combined activity of pyrethroid and PBO maintained mosquito mortality despite existing pyrethroid resistance, thereby slowing fixation of pyrethroid-resistant alleles while gradually selecting for PBO-associated resistance. Because PBO decayed faster than the pyrethroid component, its synergistic effect diminished over time, allowing pyrethroid selection to gradually increase during the later years of deployment. Nevertheless, this strategy reduced homozygous-resistant frequencies by 65.2% (95% CI 64.3–66.1%) in *An. funestus* and 79.2% (95% CI 76.6–81.8%) in *An. arabiensis* relative to continuous standard pyrethroid-only ITNs deployment by year nine. It also reduced clinical malaria incidence by 40.5% (95% CI 38.2–46.1%) and prevalence by 46.0% (95% CI 34.0–55.0%) (Fig. 9, blue curve).

#### Transitioning from standard pyrethroid-only ITNs to pyrethroid-PBO ITNs and then IG2 ITNs

Sequential deployment of standard pyrethroid-only ITNs, pyrethroid-PBO, and IG2 ITNs (Fig. 8d) produced the weakest overall selection for pyrethroid resistance. Following the initial selection for pyrethroid resistance, deployment of PBO ITNs reduced pyrethroid selection, while subsequent introduction of IG2 shifted selection towards the chlorfenapyr resistance locus (*c*). Because IG2 combines pyrethroid with chlorfenapyr, mosquitoes had to survive both insecticidal components to reproduce, reducing the selective advantage associated with pyrethroid resistance alone. In addition, the longer persistence of chlorfenapyr relative to PBO maintained mosquito mortality later in the deployment cycle, further limiting pyrethroid resistance evolution. This strategy achieved the largest reductions in homozygous-resistant mosquitoes, 71.9% (95% CI 70.8–72.9%) in *An. funestus* and 81.4% (95% CI 78.7–84.1%) in *An. arabiensis* by year 9. Correspondingly, it reduced clinical malaria incidence by 63.0% (95% CI 51.0–74.5%) and prevalence by 76.0% (95% CI 66.5–89.2%) relative to continued use of standard pyrethroid-only ITNs (Fig. 9, olive curve).

Across all deployment strategies, resistance evolved more rapidly in *An. funestus* than in *An. arabiensis*, reflecting greater exposure of the more endophilic species to ITNs. Overall, rotating insecticides with different modes of action redistributed selection pressure across the pyrethroid (*a*), PBO (*b*), and chlorfenapyr (*c*) resistance loci, preventing prolonged selection on any single resistance mechanism and thereby delaying resistance fixation while maintaining higher vector control efficacy.

## Discussion

Malaria transmission in many areas in Tanzania is seasonal and characterized by high levels of insecticide resistance [42]. While previous modelling studies have improved understanding of malaria control and insecticide resistance management, many overlook the interactions among key factors, including multiple vector species, species-specific vector dynamics, seasonality, and resistance dynamics [24–28]. This study integrated seasonal transmission, species-specific population dynamics of *An. funestus* and *An. arabiensis*, and insecticide resistance using an agent-based modelling framework (EMOD) to evaluate the impact of standard pyrethroid-only, pyrethroid-PBO, and IG2 ITNs, together with sequential deployment strategies, on malaria transmission and resistance dynamics.

In seasonal malaria transmission settings, deploying interventions such as ITNs at the onset of the peak transmission season is considered an effective strategy for maximizing their impact [26, 55]. Consistent with this, our model predicts that distributing ITNs in March, immediately before peak transmission, optimizes malaria control. Among the interventions evaluated, IG2 ITNs consistently produced the greatest reductions in malaria incidence, prevalence, and EIR, followed by pyrethroid-PBO ITNs, whereas standard pyrethroid-only ITNs were the least effective. The improved performance of pyrethroid-PBO ITNs over standard pyrethroid-only ITNs likely reflects the synergistic effect of PBO in restoring pyrethroid susceptibility, while the superior efficacy of IG2 ITNs is consistent with studies demonstrating the advantages of dual-active ingredient ITNs in areas of high pyrethroid resistance [44, 56–58]. Although both *An. funestus* and *An. arabiensis* responded to new-generation ITNs, *An. funestus* not only showed greater reductions in malaria transmission and EIR but also experienced stronger selection for insecticide resistance, consistent with its dominant role in malaria transmission in high-resistance settings [41, 42, 59]. Nevertheless, malaria incidence, prevalence, and EIR increased across all intervention scenarios by the third year, highlighting that ITNs alone are unlikely to sustain low malaria transmission and reinforcing the need for integrated vector management strategies alongside the deployment of next-generation ITNs.

Although long-lasting IRS insecticides are available [60], implementation is hindered by high costs and the disruptive nature of annual applications [61]. Consequently, IRS has been phased out in Tanzania in favour of pyrethroid-PBO ITNs [22] despite limited evidence supporting their ability to fully replace IRS in high-transmission and resistance areas [22]. While pyrethroid-PBO ITNs can avert twice as many malaria cases and help manage insecticide resistance [26], the added benefit conferred by PBO typically lasts for no more than two years [62]. This study shows that combining pyrethroid-PBO ITNs with IRS applied once every three years can effectively prevent malaria resurgence associated with declining ITN efficacy, representing a promising strategy for the NMCP to accelerate progress towards a malaria-free Tanzania [19]. Despite concerns about the feasibility of combined use in some settings [14, 63], evidence supports substantial health benefits from integrating IRS with ITNs [63, 64]. The shifting IRS from annual to triennial application may offer a cost-saving advantage. Although triennial IRS strategy has not yet been implemented in practice, field studies are recommended to assess its feasibility and effectiveness.

Repeated deployment of insecticides with the same mode of action is considered a major driver of insecticide resistance evolution [65, 66]. Consistent with previous studies [3, 13, 25, 41], the current model with its simplified resistance representation shows that continuous use of standard pyrethroid-only ITNs imposes the strong selection for pyrethroid resistance, whereas transitioning from standard ITNs to pyrethroid-PBO and subsequently IG2 ITNs redistributes selection pressure across different resistance loci, delaying the accumulation of pyrethroid-resistant genotypes while improving malaria control. These findings highlight the importance of deploying ITNs with different modes of action to mitigate resistance and reduce malaria incidence and prevalence. Notably, incorporating IG2 ITNs into the transition strategy produced the greatest reduction in both resistance development and malaria burden. This supports the strategic deployment of next-generation ITNs, particularly in high-transmission and high-resistance settings such as the lake zone of Tanzania. However, these findings should be interpreted as comparative assessments of alternative deployment strategies rather than predictions of the complete molecular or quantitative-genetic trajectory of insecticide resistance evolution.

This study contributes to the growing body of evidence supporting malaria control, elimination, and insecticide resistance management in Tanzania. Nevertheless, several limitations should be acknowledged. First, insecticide resistance was represented using a simplified multi-locus framework in which resistance to pyrethroids, PBO, and chlorfenapyr was modelled independently. Although this approach captures insecticide-specific selection and resistance dynamics during transitions between ITN types, it does not explicitly represent the full quantitative-genetic architecture of resistance, including interactions among target-site, metabolic, cuticular, and other resistance mechanisms, nor does it account for the emergence of new resistance alleles or compensatory mutations over time. Although EMOD [24] can represent a more explicit polygenic architecture through multiple loci per insecticide chemistry and genotype-specific insecticide-effect modifiers, implementing a quantitative-genetic framework such as a Polygenic Resistance Score [27] would require numerous poorly constrained assumptions about resistance inheritance, genotype-to-phenotype relationships, fitness costs, cross-resistance, exposure heterogeneity, and mosquito migration. Given the limited empirical data available to parameterize these processes, multiple parameter combinations could plausibly reproduce similar phenotypic mortality or bioassay survival patterns. We therefore adopted a parsimonious and transparent modelling framework that is well suited for comparing alternative ITN deployment strategies over a more complex model that could imply unwarranted precision. Second, the model primarily captures the lethal effects of ITNs and does not explicitly incorporate non-lethal effects, such as deterrence, excito-repellency, altered host-seeking behaviour, or sublethal impacts on mosquito survival and reproduction, all of which may also influence malaria transmission and resistance evolution. Third, the model considers only *Plasmodium falciparum*; however, this is unlikely to materially affect the findings because this parasite accounts for more than 96% of malaria infections in mainland Tanzania [19]. Finally, the model does not account for resistance selection arising from agricultural insecticide use or other environmental exposures. Consequently, the findings should be interpreted within the assumptions and calibration of the model and extrapolated to other epidemiological settings with appropriate caution.

Despite these limitations, one-at-a-time sensitivity analyses showed that the principal conclusions were robust to plausible variation in the parameters presented in Table 1. Across the tested ranges, repeated deployment of standard pyrethroid-only ITNs consistently imposed the strongest selection for pyrethroid resistance, whereas transitioning to pyrethroid-PBO and subsequently IG2 ITNs continued to reduce both malaria burden and pyrethroid-resistant genotype frequencies. The pyrethroid resistance killing modifier was the most influential parameter affecting resistance selection, while the IRS initial killing effect had the greatest influence among the IRS parameters. Although the one-at-a-time approach does not capture higher-order interactions among parameters, it provides confidence that the main conclusions are not driven by individual parameter choices (Supplementary Material). To our knowledge, this is the first modelling study to evaluate new-generation ITNs while simultaneously incorporating seasonal transmission, species-specific dynamics of *An. funestus* and *An. arabiensis*, and a multi-locus representation of resistance to pyrethroids, PBO, and chlorfenapyr.

## Conclusions

The effective malaria control in Tanzania, particularly in high-transmission zones and resistant areas, requires integrated, and adaptive vector control tools and strategies. This study indicates that continuous use of pyrethroid-only ITNs maintains strong selection pressure and reduces the impact of these interventions on malaria transmission. In contrast, transitioning or rotating ITNs with diverse modes of action, together with periodic use of IRS, effectively mitigates insecticide resistance and supports long-term malaria control.

## Supplementary Information

Supplementary Material 1.

## Data Availability

All data supporting the findings of this study are included within this article.

## Appendix

See Table 1.

**Table 1:** Key parameters and values used for simulations

Parameter	Description	Value and source
ITN initial killing effect	Probability that a susceptible mosquito is killed when attempting to feed on an ITN-protected human, effect decays over time	93% [59], 98% [67], and 98.7% [68] for standard pyrethroid-only, pyrethroid-PBO, and IG2 ITNs, respectively
Killing decay time constant	Exponential decay time (days) of the initial killing effect	1420 days for pyrethroid and 1260 days for PBO (derived from assumed half-lives of 2.7 and 2.4 years, respectively [69]); and 1580 days for chlorfenapyr (derived based on the evidence from Tanzania demonstrating the protective efficacy of IG2 for approximately 3 years [70]), 90 days for IRS (adopted from WHO operational manual [71])
IRS initial killing effect	Probability that a susceptible mosquito is killed upon contacting a treated indoor surface, effect decays over time	99% [62]
ITN initial blocking effect	Probability that an ITN blocks a mosquito's attempt to feed on a protected human indoors, effect decays over time	90% (adopted from [72]); 92% [73]; 93.2% (derived from [74]), for standard pyrethroid-only, pyrethroid-PBO, and IG2 ITNs, respectively
ITN blocking decay time constant	Exponential decay time (days) of the ITN initial blocking effect	730 days (adopted from [72])
Indoor feeding/resting fraction	Fraction of mosquito feeding/ resting occurring indoors	63.1% for *An. arabiensis,* 78.2% for *An. funestus* [46]
Anthropophilic indices	Proportion of blood-fed mosquitoes with human blood meals	44%[47] for *An. arabiensis,* 64% [48] for *An. funestus*
Mosquito life expectancy	Baseline number of days an adult mosquito survives	20 days for *An. arabiensis* and 25 days for *An. funestus* (the *An. arabiensis* value was adopted from the EMOD documentation [49], whereas the *An. funestus* value was assumed based on evidence from Tanzania indicating a longer adult lifespan than *An. arabiensis* [75])
Standard pyrethroid-only ITN resistance mortality in *An. funestus*	*An. funestus* genotype-specific mortality associated with standard pyrethroid-only ITN	Heterozygous: 50% (Estimated from genotype-specific survival counts in [39])
Standard pyrethroid-only ITN resistance mortality in *An. arabiensis*	*An. arabiensis* genotype-specific mortality associated with standard pyrethroid-only ITN	Heterozygous: 51% (Estimated from genotype-specific survival counts in [76, 77])
Pyrethroid-PBO ITN resistance mortality in *An. funestus*	*An. funestus* genotype-specific mortality associated with pyrethroid-PBO ITN	Heterozygous: 67% (extracted from [78])
Pyrethroid-PBO ITN resistance mortality in *An. arabiensis*	*An. arabiensis* genotype-specific mortality associated with pyrethroid-PBO ITN	Heterozygous: 71.5% (Assumed from phenotypic resistance in [38]
IG2 ITN resistance mortality in *An. funestus*	*An. funestus* genotype-specific mortality associated with IG2 ITN	Heterozygous: 73% (Assumed from phenotypic resistance in [79]
IG2 ITN resistance mortality in *An. arabiensis*	*An. arabiensis* genotype-specific mortality associated with IG2 ITN	Heterozygous: 80% (Assumed from phenotypic resistance in [80])
IRS resistance mortality	Mosquito genotype-specific mortality associated with IRS	Heterozygous: 83% (Assumed from phenotypic resistance [77])
ITN and IRS useful lifetime	Recommended duration of effectiveness under field conditions before replacement or insecticide retreatment	3 × 365 days for ITNs [14], 365 days for IRS[20]

Details of the derivation, calculations, and estimation procedures used to obtain the parameter values are provided in supplementary material (Tables SA1 and SA2)

## Abbreviations

ITNs: Insecticide-treated nets
EMOD: Epidemiological modeling software
CI: Confidence interval
IRS: Indoor residual spraying
WHO: World Health Organization
NMCP: National Malaria Control Programme
PBO: Pyrethroid-piperonyl butoxide
