# Supplementary material for: Modelling the impact of adopting new-generation insecticide-treated nets on malaria transmission and insecticide resistance
Source: Parasit Vectors. 2026 Aug 14;19:429. doi: 10.1186/s13071-026-07611-x (PMC13633644; doi:10.1186/s13071-026-07611-x)
Supplement: Supplementary file 1 [file 13071_2026_7611_MOESM1_ESM.docx]

**Modelling the impact of adopting new-generation insecticide-treated nets on malaria transmission and insecticide resistance**

Hamenyimana E. Gervas, Maranya M. Mayengo, Frank Chacky, Yeromin P. Mlacha, Halfan S. Ngowo, Fredros O. Okumu & Prashanth Selvaraj

*Detailed description on calculation/estimation of homozygous and heterozygous genotype values.*

Most of the estimated mortalities corresponding to heterozygous genotype frequencies for ITNs and IRS were calculated using Equation (1), adapted from WHO guidelines [1], with homozygous mortality assumed to be half of heterozygous mortality. The estimated values represent the genotype-specific mortality for the interventions and were used as killing modifiers in EMOD to model resistance in homozygous and heterozygous mosquitoes. Although derived from experimental or field data, these estimates are context-specific approximations, should not be considered universal constants or definitive guidelines. Refer Table SA1 for descriptions on how was calculated and estimated.

Mortality = $\frac{Number of dead mosquitoes}{Total sample size (alive+dead mosquitoes)}$ $\times100\%$	(1)

*Table SA1: Procedures used to calculate and estimate model parameter values, including genotype-specific parameters for homozygous and heterozygous mosquitoes. Homozygous mosquitoes were assumed to experience a mortality rate equal to half that of heterozygous mosquitoes.*

| Parameter | Value & source | Explanation/how derived or estimated |
| --- | --- | --- |
| Killing decay time constant | 1420 days for pyrethroid and 1260 days for PBO (derived from assumed half-lives of 2.7 and 2.4 years, respectively [2] ) | Using the half-life for pyrethroid killing (*h*)=2.7 years [2],  Decay time constant= $\frac{h}{ln(2)}$ = $\frac{2.7}{ln(2)}$ ≈ 1420 days,  For PBO, Decay time constant= $\frac{h}{ln(2)}$ = $\frac{2.4}{ln(2)}$ ≈ 1260 days, |
|  |  | Based on the evidence from [3], we assume the half-life for chlorfenapyr killing (*h*) = 3 years,  Decay time constant= $\frac{h}{ln(2)}$ = $\frac{1095}{ln(2)}$ ≈ 1580 days |
| ITN initial blocking effect | 93.2 % (derived from [4]) for IG2 ITNs | Blocking effect for IG2 ITNs ≈ $\frac{89.09\% + 90.38\% + 100\%}{3}$ = 93.2% |
| Mosquito life expectancies | 20 days for An. arabiensis and 25 days for An. funestus (the An. arabiensis value was adopted from the EMOD documentation [5], whereas the An. funestus value was assumed based on evidence from Tanzania indicating a longer adult lifespan than An. arabiensis [6]). | In EMOD, adult life expectancy is a baseline survival parameter; with vector aging enabled, age-dependent senescence results in a realized mean adult lifespan shorter than the specified value (Specifically, adult life expectancy equals to 20 days corresponds to a mean adult female mosquito lifespan of approximately 9-9.5 days [5]). The senescence function in EMOD follows the model described by Styer et al. [7]. A value of 25 days was assumed for *An. funestus* based on evidence from Tanzania indicating a longer adult lifespan than *An. arabiensis* [6]. |
| Standard pyrethroid-only ITN resistance mortality in *An. funestus* | Heterozygous: 50.0% (Calculated from genotype-specific survival counts in [8]) | From [8]:  Heterozygous RS dead= 23,  Heterozygous RS alive = 23, then  mortality = $\frac{23}{23+23}$×100%= 50%. |
|  |  | Homozygous mortality= $\frac{1}{2}$× 50% =25% |
| Standard pyrethroid-only ITN resistance mortality in *An. arabiensis* | Heterozygous: 51%  Calculated from genotype-specific survival counts in [9,10]. | From [9],  Heterozygous RS alive= 5, Heterozygous RS dead = 15, then  mortality = $\frac{15}{15+5}$×100%= 75%.  From [10],  Heterozygous RS alive= 63, Heterozygous RS dead = 22, then  mortality = $\frac{22}{63+22}$×100%= 26%.  Thus, heterozygous mortality = $\frac{75 \%+26 \%}{2}$ = 50.5 % ≈ 51 % |
|  |  | Homozygous mortality= $\frac{1}{2}$× 51% =25.5% |
| Pyrethroid-PBO ITN resistance mortality in *An. funestus* | Heterozygous: 67% (extracted from phenotypic resistance in [11], because all resistant mosquitoes were heterozygous | Homozygous mortality= $\frac{1}{2}$× 67% =33.5% |
| Pyrethroid-PBO ITN resistance mortality in *An. arabiensis* | Heterozygous: 71.5% (Assumed from phenotypic resistance in Figure 5 in [12]) | From Figure 5 (Kakonko and Tandahimba): Mortality= $\frac{75+68}{2}$=71.5% |
|  |  | Homozygous mortality= $\frac{1}{2}$× 71.5% =35.8% |
| IG2 ITN resistance mortality in *An. funestus* | Heterozygous: 73% (Assumed from phenotypic resistance in Figure 3 in [13] | Homozygous mortality= $\frac{1}{2}$× 73% =36.5% |
| IG2 ITN resistance mortality in *An. arabiensis* | Heterozygous: 80% (Assumed from phenotypic resistance in [14]) | Homozygous mortality= $\frac{1}{2}$× 80%=40% |
| IRS resistance mortality | Heterozygous: 83% (Assumed from phenotypic resistance from Table 1 in [10]) | Homozygous mortality= $\frac{1}{2}$× 83%=41.5% |

*Estimation of mortality associated with the PBO-mediated synergistic effect and chlorfenapyr*

To investigate resistance selection under continuous deployment of standard pyrethroid-only ITNs and subsequent transitions to next-generation ITNs, insecticide resistance was represented using a simplified multi-locus framework. Resistance to each insecticide chemistry was modelled independently by assigning a separate locus to each active component: locus *a* for pyrethroid resistance, locus *b* for the effectiveness of the PBO synergist, and locus *c* for chlorfenapyr effectiveness. Consequently, pyrethroid-PBO ITNs act through loci *a* and *b*, whereas Interceptor® G2 (IG2) ITNs act through loci *a* and *c*. Selection at each locus was assumed to occur independently; therefore, resistance to one insecticide chemistry did not confer resistance to the others. Each locus comprised three genotype classes (homozygous susceptible, heterozygous, and homozygous resistant), with heterozygotes assumed to exhibit partial resistance and the two homozygotes representing fully susceptible and fully resistant phenotypes, respectively [8,12,15,16].

Because pyrethroid-PBO ITNs combine a pyrethroid insecticide with the PBO synergist, whereas Interceptor® G2 (IG2) ITNs combine a pyrethroid insecticide with chlorfenapyr, the overall mosquito mortality was assumed to result from the independent contributions of the two active components [17]. Under this assumption, and following the Bliss independent-action principle [18] together with the probability rule for the union of two independent events [19], the probability of mosquito mortality induced by the ITN was calculated as the combined probability of mortality attributable to the two independently acting insecticide components. Accordingly, the overall mortality induced by a new-generation ITN was estimated as:

K_ITN_ = 1- ((1-K_A_ ×M_a_) (1-K_B_ × M_b_))	(1)

where,

- K_ITN_ is the overall mosquito mortality induced by the ITN (pyrethroid-PBO or IG2);
- K_A_ is the mortality attributable to the pyrethroid component;
- K_B_ is the mortality attributable to the PBO-mediated synergistic effect (for pyrethroid-PBO ITNs) or chlorfenapyr (for IG2 ITNs);
- M_a_ and M_a_ are genotype-specific susceptibility modifiers for the pyrethroid and second active component, respectively. For fully susceptible mosquitoes, both modifiers were assumed to equal 1.

The resulting parameter values presented in Table SA2

*Table SA2: Procedures used to calculate the mortality attributable to the PBO-mediated synergistic effect and chlorfenapyr.*

| ITN | How derived/calculated/estimated | |
| --- | --- | --- |
| Pyrethroid-PBO ITN | From Table 1:  Overall mortality (K_ITN_) = 0.98  Mortality due to pyrethroid (K_A_) = 0.93,  Mortality due PBO synergist effect, K_B_ = ? | K_ITN_ = 1- ((1-K_A_ × M_a_) (1-K_B_ ×M_b_))  ⇒0.98 =1- ((1- 0.93×1) × (1- K_B_×1))  ⇒ K_B_ = 0.71 |
| IG2 ITN | From Table 1:  Overall mortality (K_ITN_) = 0.987  Mortality due to pyrethroid (K_A_) = 0.93,  Mortality due chlorfenapyr, K_B_ = ? | K_ITN_ = 1- ((1-K_A_ × M_a_) (1-K_B_ ×M_b_))  ⇒0.987 =1- ((1- 0.93×1) × (1- K_B_×1))  ⇒ K_B_ = 0.81 |

These genotype-specific mortality parameters were used to define aggregate resistance phenotypes for the purposes of scenario comparison. They were not intended to estimate the underlying molecular architecture of resistance. A more detailed polygenic EMOD implementation would require additional longitudinal genotype, phenotype, and fitness-cost data that were not available for the present analysis.

**Sensitivity analysis**

We conducted one-at-a-time (OAT) sensitivity analyses to assess how the model outcomes respond to uncertainty in the parameters listed in Table 1. Each parameter was varied independently by multiplying its nominal value by 0.6, 0.8, 1.2, and 1.4 (that is, by ±20% and ±40%), while all other parameters were held at their nominal values; parameters representing probabilities or fractions were capped at 1.0. Two ITN deployment scenarios were analysed: repeated deployment of standard pyrethroid-only ITNs (STD to STD to STD), and the sequential transition from standard ITNs to pyrethroid-PBO ITNs and then to IG2 ITNs (STD to PBO to IG2). For each scenario we recorded the year-9 frequency of homozygous pyrethroid-resistant *An. funestus* and *An. arabiensis*, the year-9 prevalence of infection in children under five years of age, and the cumulative clinical malaria incidence in children under five over the nine-year period, using ten stochastic replicates per parameter combination. Because Table 1 also includes IRS parameters, we additionally performed a focused OAT analysis of the three IRS-specific parameters (initial killing effect, killing decay constant, and resistance killing modifier) for a pyrethroid-PBO ITN programme combined with IRS deployed in year 2 of each three-year cycle, using fifty stochastic replicates.

Under continuous standard pyrethroid-only ITNs (Figure SA1), the pyrethroid resistance killing modifier was by far the most influential parameter for the selection of pyrethroid resistance in both vector species, while year-9 malaria incidence in children under five was most sensitive to the *An. funestus* indoor feeding fraction and the pyrethroid initial blocking effect. Under the sequential STD to PBO to IG2 transition (Figure SA2), the pyrethroid resistance killing modifier again dominated the residual year-9 pyrethroid-resistance frequency, whereas cumulative malaria incidence was governed primarily by the shared net-efficacy and vector-behaviour parameters; the PBO-synergist and chlorfenapyr parameters had progressively smaller effects, consistent with the later deployment of PBO and IG2 ITNs. An. *arabiensis* showed the same qualitative pattern at a lower absolute resistance frequency (Table SA1). In the IRS analysis (Table SA2), the IRS initial killing effect was the dominant IRS parameter, whereas the IRS killing decay constant and resistance killing modifier produced smaller changes that were comparable to the stochastic uncertainty of this low-transmission setting. Across all perturbations the main qualitative conclusions of the study were preserved: continuous standard pyrethroid-only ITNs maintained strong pyrethroid-resistance selection, whereas the transition to PBO and then IG2 ITNs continued to reduce both malaria burden and pyrethroid-resistant genotype frequencies.

*Figure SA1. One-at-a-time sensitivity analysis under repeated standard pyrethroid ITN deployment (STD to STD to STD). Each horizontal bar shows the range of the year-9 outcome when the corresponding Table 1 parameter is varied between 0.6 and 1.4 times its nominal value, with all other parameters held fixed. Orange indicates the direction of parameter increase and blue the direction of decrease; the dashed vertical line marks the nominal value. Left: frequency of homozygous pyrethroid-resistant An. funestus (locus a); centre: frequency of homozygous pyrethroid-resistant An. arabiensis (locus a); right: annual malaria incidence in children under five years.*

*Figure SA2. One-at-a-time sensitivity analysis under the sequential STD to PBO to IG2 ITN transition. Bars and colours are as in Figure SA1, with the PBO-synergist and chlorfenapyr (IG2) net parameters included in addition to the standard-net and vector parameters. Left: year-9 frequency of homozygous pyrethroid-resistant An. funestus (locus a); right: cumulative malaria incidence in children under five over years 1 to 9 (cases per child).*

*Table SA3. Sensitivity of the year-9 An. arabiensis homozygous pyrethroid-resistant frequency and the year-9 prevalence of infection in children under five to the Table 1 parameters, under the sequential STD to PBO to IG2 transition. For each parameter the nominal value and the range obtained across the 0.6 to 1.4 perturbations are shown. Under this transition the An. arabiensis homozygous pyrethroid-resistant frequency remains very low (below about 0.013) and close to the stochastic detection limit, so small absolute differences between parameters should be interpreted with caution.*

| **Parameter** | ***An. arabiensis* resistance (nominal)** | ***An. arabiensis* resistance (range)** | **u5 prevalence (nominal)** | **u5 prevalence (range)** |
| --- | --- | --- | --- | --- |
| *An. arabiensis* adult lifespan | 0.0099 | 0.0000 to 0.0099 | 0.228 | 0.175 to 0.394 |
| *An. funestus* adult lifespan | 0.0099 | 0.0000 to 0.0124 | 0.228 | 0.162 to 0.584 |
| *An. arabiensis* anthropophily | 0.0099 | 0.0051 to 0.0109 | 0.228 | 0.173 to 0.456 |
| *An. funestus* anthropophily | 0.0099 | 0.0045 to 0.0099 | 0.228 | 0.119 to 0.331 |
| An. arabiensis indoor feeding fraction | 0.0099 | 0.0045 to 0.0099 | 0.228 | 0.056 to 0.515 |
| *An. funestus* indoor feeding fraction | 0.0099 | 0.0070 to 0.0099 | 0.228 | 0.000 to 0.558 |
| Pyrethroid initial killing effect | 0.0099 | 0.0046 to 0.0099 | 0.228 | 0.173 to 0.566 |
| Pyrethroid initial blocking effect | 0.0099 | 0.0055 to 0.0099 | 0.228 | 0.000 to 0.557 |
| Pyrethroid killing decay τ (days) | 0.0099 | 0.0045 to 0.0107 | 0.228 | 0.228 to 0.513 |
| Pyrethroid blocking decay τ (days) | 0.0099 | 0.0067 to 0.0099 | 0.228 | 0.115 to 0.514 |
| ITN useful lifetime (days) | 0.0099 | 0.0053 to 0.0131 | 0.228 | 0.228 to 0.512 |
| Pyrethroid resistance killing modifier | 0.0099 | 0.0019 to 0.0396 | 0.228 | 0.227 to 0.459 |
| PBO synergist initial killing effect | 0.0099 | 0.0071 to 0.0099 | 0.228 | 0.228 to 0.401 |
| PBO synergist killing decay τ (days) | 0.0099 | 0.0079 to 0.0100 | 0.228 | 0.228 to 0.454 |
| Chlorfenapyr initial killing effect | 0.0099 | 0.0079 to 0.0103 | 0.228 | 0.171 to 0.229 |
| Chlorfenapyr killing decay τ (days) | 0.0099 | 0.0090 to 0.0099 | 0.228 | 0.114 to 0.229 |
| PBO synergist resistance killing modifier | 0.0099 | 0.0082 to 0.0099 | 0.228 | 0.171 to 0.345 |
| Chlorfenapyr resistance killing modifier | 0.0099 | 0.0081 to 0.0105 | 0.228 | 0.172 to 0.233 |

*Table SA4. Sensitivity of cumulative under-five malaria incidence (clinical cases per child, years 1 to 9) to the three IRS-specific Table 1 parameters, for a pyrethroid-PBO ITN programme with IRS deployed in year 2 of each three-year cycle. Each parameter was varied independently between 0.6 and 1.4 times its nominal value (fifty stochastic replicates per cell); the nominal value is 1.79 cases per child. The IRS initial killing effect is capped at 1.0, so its 1.2 and 1.4 columns coincide.*

| **IRS parameter** | **0.6** | **0.8** | **1.0 (nominal)** | **1.2** | **1.4** |
| --- | --- | --- | --- | --- | --- |
| IRS initial killing effect | 2.89 | 2.41 | 1.79 | 1.57 | 1.57 |
| IRS killing decay constant | 2.13 | 2.34 | 1.79 | 2.07 | 1.30 |
| IRS resistance killing modifier | 1.74 | 1.84 | 1.79 | 2.24 | 2.51 |

**Reference:**

1. WHO. Test procedures for insecticide resistance monitoring in malaria vector mosquitoes. World Health Organization. Geneva:World Health Organization; 2018.

2. Champagne C, Lemant J, Assenga A, Kibondo UA, Lekundayo RG, Mbuba E, et al. Cascades of effectiveness of next-generation insecticide-treated nets against malaria, from entomological trials to real-life conditions. Nat Commun. 2025; 16:11162-.

3. Mosha JF, Matowo NS, Kulkarni MA, Messenger LA, Lukole E, Mallya E, et al. Effectiveness of long-lasting insecticidal nets with pyriproxyfen–pyrethroid, chlorfenapyr–pyrethroid, or piperonyl butoxide–pyrethroid versus pyrethroid only against malaria in Tanzania: final-year results of a four-arm, single-blind, cluster-randomised…. Lancet Infect Dis. 2024; 24:87–97.

4. Zoungbédji DM, Padonou GG, Sovi A, Konkon AK, Salako AS, Azondékon R, et al. Bio-efficacy of Olyset® Plus, PermaNet® 3.0 and Interceptor® G2 on pyrethroid-resistant populations of *Anopheles gambiae* s.l. prior to the June 2023 net distribution campaign in Benin, West Africa. Trop Med Health. 2024;52:34-.

5. Institute for Disease Modeling (IDM). EMOD Documentation: Vector Life Cycle (emodpy-malaria). 2026. https://emod.idmod.org/emodpy-malaria/emod/parameter-configuration-vector-lifecycle/

6. Ntabaliba W, Vavassori L, Stica C, Makungwa N, Odufuwa OG, Swai JK, et al. Life expectancy of *Anopheles funestus* is double that of *Anopheles arabiensis* in southeast Tanzania based on mark-release-recapture method. Scientific Reports. 2023;13:1–9.

7. Styer LM, Minnick SL, Sun AK, Scott TW. Mortality and reproductive dynamics of Aedes aegypti (Diptera: Culicidae) fed human blood. Vector-Borne and Zoonotic Diseases. 2007;7:86–98.

8. Djoko Tagne CS, Kouamo MFM, Tchouakui M, Muhammad A, Mugenzi LJL, Tatchou-Nebangwa NMT, et al. A single mutation G454A in the P450 CYP9K1 drives pyrethroid resistance in the major malaria vector *Anopheles funestus* reducing bed net efficacy. Genetics. 2025. 229.

9. Messenger LA, Impoinvil LM, Derilus D, Yewhalaw D, Irish S, Lenhart A. A whole transcriptomic approach provides novel insights into the molecular basis of organophosphate and pyrethroid resistance in *Anopheles arabiensis* from Ethiopia. Insect Biochem Mol Biol. 2021; 139:103655.

10. Messenger LA, Shililu J, Irish SR, Anshebo GY, Tesfaye AG, Ye-Ebiyo Y, et al. Insecticide resistance in *Anopheles arabiensis* from Ethiopia (2012-2016): A nationwide study for insecticide resistance monitoring. Malar J. 2017; 16:1–14.

11. Riveron JM, Chiumia M, Menze BD, Barnes KG, Irving H, Ibrahim SS, et al. Rise of multiple insecticide resistance in *Anopheles funestus* in Malawi: A major concern for malaria vector control. Malar J. 2015; 14:1–9.

12. Kabula B, Mlacha YP, Serbantez N, Nhiga SL, Mkude S, Kiware S, et al. Pyrethroid-resistant malaria vector *Anopheles gambiae* restored susceptibility after pre-exposure to piperonyl-butoxide: results from country-wide insecticide resistance monitoring in Tanzania, 2023. Malar J. 2024; 23:1–16.

13. Tungu PK, Michael E, Sudi W, Kisinza WW, Rowland M. Efficacy of interceptor® G2, a long-lasting insecticide mixture net treated with chlorfenapyr and alpha-cypermethrin against *Anopheles funestus*: experimental hut trials in north-eastern Tanzania. Malar J. 2021;20.

14. Tchouakui M, Assatse T, Tazokong HR, Oruni A, Menze BD, Nguiffo-Nguete D, et al. Detection of a reduced susceptibility to chlorfenapyr in the malaria vector *Anopheles gambiae* contrasts with full susceptibility in *Anopheles funestus* across Africa. Sci Rep. 2023;13.

15. Selvaraj P, Wenger EA, Bridenbecker D, Windbichler N, Russell JR, Gerardin J, et al. Vector genetics, insecticide resistance and gene drives: An agent-based modeling approach to evaluate malaria transmission and elimination. PLoS Comput Biol. 2020;16.

16. Collins E, Vaselli NM, Sylla M, Beavogui AH, Orsborne J, Lawrence G, et al. The relationship between insecticide resistance, mosquito age and malaria prevalence in *Anopheles gambiae* s.l. from Guinea. Sci Rep. 2019; 9:1–12.

17. WHO. WHO guidelines for malaria. Geneva: World Health Organization; 2023.

18. Bliss Ci. The toxicity of Poisons applied jointly. Annals of Applied Biology. 1939; 26:585–615.

19. Doyle P. Grinstead and Snell’s Introduction to Probability. 2006.
